# Effect of Exercising with Others on Incident Functional Disability and All-Cause Mortality in Community-Dwelling Older Adults: A Five-Year Follow-Up Survey

**DOI:** 10.3390/ijerph17124329

**Published:** 2020-06-17

**Authors:** Yuya Fujii, Keisuke Fujii, Takashi Jindo, Naruki Kitano, Jaehoon Seol, Kenji Tsunoda, Tomohiro Okura

**Affiliations:** 1 Doctoral Program in Physical Education, Health and Sport Sciences, University of Tsukuba, Tsukuba 305-8577, Japan; 2 Japan Society for the Promotion of Science, Tokyo 102-0083, Japan; 3 Faculty of Health Sciences, Kansai University of Health Sciences, Osaka 590-0482, Japan; k.fujii@kansai.ac.jp; 4 Physical Fitness Research Institute, Meiji Yasuda Life Foundation of Health and Welfare, Tokyo 192-0001, Japan; ta-jindo@my-zaidan.or.jp (T.J.); na-kitano@my-zaidan.or.jp (N.K.); 5 R&D Center for Tailor-Made QOL, University of Tsukuba, Tsukuba 305-8550, Japan; seol.jaehoon.ge@u.tsukuba.ac.jp; 6 Faculty of Social Welfare, Yamaguchi Prefectural University, Yamaguchi 753-8502, Japan; ktsunoda@yamaguchi-pu.ac.jp; 7 Faculty of Health and Sport Sciences, University of Tsukuba, Tsukuba 305-8577, Japan; okura.tomohiro.gp@u.tsukuba.ac.jp

**Keywords:** physical activity, long-term care, social networks, death and dying

## Abstract

We clarified the effect of exercising with others on the risks of incident functional disability and all-cause mortality among community-dwelling adults. We used an inventory mail survey with a five-year follow-up for 1520 independently living older adults (mean age: 73.4 ± 6.3 years) in Kasama City, Japan. Subjects responded to a self-reported questionnaire in June 2014. Exercise habits and the presence of exercise partners were assessed. Subjects were classified into three groups: Non-exercise, exercising alone, and exercising with others. Follow-up information and date of incident functional disability and death during the five-year follow-up were collected from the database. To compare the association between exercise habits and functional disability and mortality, Cox regression analysis was conducted. Compared with the non-exercise group, exercising with others had significantly lower hazard ratios (HRs) for functional disability (0.59, 95% confidence interval (CI) 0.40–0.88) and mortality (0.40, 95% CI 0.24–0.66) in the covariate models. Compared with exercising alone, exercising with others decreased the HRs for incident functional disability (0.53, 95% CI: 0.36–0.80) and mortality (0.50, 95% CI 0.29–0.85) rates in the unadjusted model; these associations were not significant in the covariate models. Exercising with others can contribute to functional disability prevention and longevity.

## 1. Introduction

Engaging in leisure time physical activity (LTPA) has many health benefits for older adults, such as reducing the risks of functional disability [1,2], mental illness [3], and cognitive decline [4]. Owing to these health benefits, LTPA is known to be positively correlated with a lower mortality rate [5,6]. Therefore, participation in LTPA, especially exercise, is strongly recommended for older adults to maintain health [7]. 

While many studies have mainly focused on the quantitative aspect of exercise such as intensity, duration, and frequency, recent studies have been focusing on the social aspect of the effect of exercise on older adults. Interestingly, a growing body of evidence has confirmed that exercising with others has more desirable health benefits than exercising alone. For example, previous studies have shown that exercising with others is correlated with a lower risk of depression [8], better subjective health status [9], lower incidence of falls [10], and higher levels of physical activity [11] than exercising alone.

However, to our knowledge, there is limited literature regarding the effect of exercising with others on robust clinical endpoints, such as functional disability and death. Only one cohort study has shown that older people who participated in sports organizations had a lower risk of functional disability over four years than those who exercised alone [12]. Based on these findings, we hypothesized that people who exercise with others have a lower risk of incident functional disability than those who do not exercise and those who exercise alone. Conversely, no prior study has investigated the relationship between exercising with others and mortality. Thus, it is still unknown whether exercising alone and exercising with others differ in their association with mortality. In modern society, since enhancing independence and longevity is important to reduce healthcare costs, it is useful to elucidate the effect of exercising with others on robust endpoints. Thus, to address the gap in knowledge, we aimed to clarify and compare the risks of functional disability and mortality between not exercising, exercising alone, and exercising with others among community-dwelling older adults. 

## 2. Materials and Methods

### 2.1. Study Design and Participants

In this study, we conducted an inventory mail survey that targeted community-dwelling older adults, defined as adults aged 65 years or older. The survey was conducted in the Iwama area of Kasama City, rural Japan. In June 2014, the baseline survey was conducted for all older adults living in the Iwama area, excluding those who received long-term care insurance (LTCI) at that time [13]. Self-reported questionnaires were sent to 3549 potential subjects; 2020 subjects returned the questionnaires (response rate: 56.9%). Subjects with incomplete responses or missing values (*n* = 500) were excluded, and the remaining 1520 older adults were included in the analysis (valid response rate: 42.8%). The follow-up information of the development of functional disability, death, or moving to another city prior to June 2019 was obtained from the municipal database.

This study was approved by the ethics committee of the University of Tsukuba (Ref No. Tai 26-31). The aim and procedure of the survey were explained in a document, and informed consent was assumed with the voluntary return of the questionnaires. 

### 2.2. Measurements and Methods

#### 2.2.1. Functional Disability and Mortality

Our primary endpoints were functional disability and death. Follow-up data of subjects were stored in Kasama City’s LTCI database, and we collected the information regarding the nature and date of incident functional disability and death from June 2014 up to June 2019.

We defined functional disability using the certification for LTCI, which is the system established by the Japanese government to objectively judge the levels of care, based on the national uniform standards and support required for older adults with functional disabilities [13]. This is an important indicator because it enables people to receive care services based on their level of needs. The eligibility for LTCI is determined by a two-step assessment process after the elderly person or his/her caregiver applies to the municipality. First, based on a visit assessment by a municipal official and an opinion from the attending doctor, the time required for the care is estimated and classified into seven levels. Then, the results of the first assessment will be reviewed by the long-term care need certification committee, comprised of specialists in public health, medical care, and social welfare, to decide the level of LTCI. The levels of LTCI certification consist of Support Levels 1 and 2 and Care Levels 1–5. In this study, Care Level 1 (i.e., difficulty in performing essential daily life activities by himself/herself) or higher represented incident functional disability. This level indicates that the person needs partial support in daily life activities due to physical or cognitive disability.

The data of those who moved to another city during the follow-up period were also collected. Of all subjects, eight (0.5%) moved out of the city without the occurrence of any endpoints. Thus, these subjects were treated as “survivors” and censored at that time point in the analysis.

#### 2.2.2. Exercise Habits

These were assessed by the following question: “Who do you usually (once a week or more) exercise with”? The response options were as follows (multiple answers allowed): “no exercise”, “exercise by oneself”, “exercise with spouse”, “exercise with same-gender friends”, “exercise with opposite-gender friends”, “exercise with son/daughter”, “exercise with grandchildren” and “exercise with exercise experts”. According to the responses, subjects were classified into three groups: Non-exercise (i.e., those who selected “not exercise”), exercising alone (i.e., those who selected “by oneself” only), and exercising with others (i.e., those who selected the latter six answers) in accordance with a previous study [11].

#### 2.2.3. Covariates

We used the variables obtained from the baseline mail survey as covariates. It included age, sex, body mass index (BMI), living arrangement, subjective economic condition, medical history, fall history, and depressive symptoms. BMI was calculated using self-reported height and weight and categorized as <18.5, 18.5–29.9, and ≥30 kg/m^2^. The accuracy of self-reported BMI had reasonably high concordance among the Japanese elderly (weighted kappa values: 0.715 and 0.670 for men and women, respectively; *p* < 0.001) [14]. Subjective economic condition was investigated by asking, “How do you think about your current economic condition?” with the following five response options: “very poor”, “poor”, “normal”, “good”, and “very good”. Medical history included the history of heart diseases, stroke, and joint pain. Fall history was assessed by asking whether the subjects had experienced one or more falls in the previous year. To assess depressive mood, this study used the mood domain of the Kihon Checklist [15], which comprises five items with binary responses (yes/no). These five items were summed to evaluate depressive mood [15] with a higher value indicative of worse depressive symptoms (range: 0–5 points). Living arrangement was categorized as living alone or living with others. Subjective economic condition was categorized as poor/very poor or normal/good/very good. Age and depressive symptoms were used as continuous variables.

#### 2.2.4. Statistical Analysis

To compare the baseline characteristics by exercise pattern, we used chi-squared tests for categorical variables and analysis of variance for continuous variables. To investigate the relationship between exercise habits and the risk of incident functional disability and mortality, we conducted Cox proportional hazards regression analysis and calculated hazard ratios (HRs) and 95% confidential intervals. The dependent variables were the occurrence of functional disability and mortality over five years, and the independent variables were exercise habits with the non-exercise group as the reference. We used three models in this study: Unadjusted model, model 1 (adjusted for age and sex), and model 2 (further adjusted for BMI, living arrangement, subjective economic condition, medical history, fall history, and depressive symptoms). Moreover, we generated Kaplan–Meier curves for incident functional disability and mortality adjusting for covariates in model 2. Additionally, for direct comparison of exercising alone and exercising with other groups, we analyzed only those who exercised using all models. All analyses were performed using IBM SPSS 25.0 (IBM Corp., Armonk, NY, USA), and the significance level was set at 5%. 

## 3. Results

Table 1 shows the baseline characteristics of participants by exercise habits. The mean age was 73.4 ± 6.3 years, and 48.8% were male. Among the subjects, 541 (35.6%), 461 (30.3%), and 518 (34.1%) subjects were not exercising, exercising alone, and exercising with others, respectively. Significant inter-group differences were found in terms of age, sex, heart disease, fall history, and depressive symptoms.

**Figure 1:**
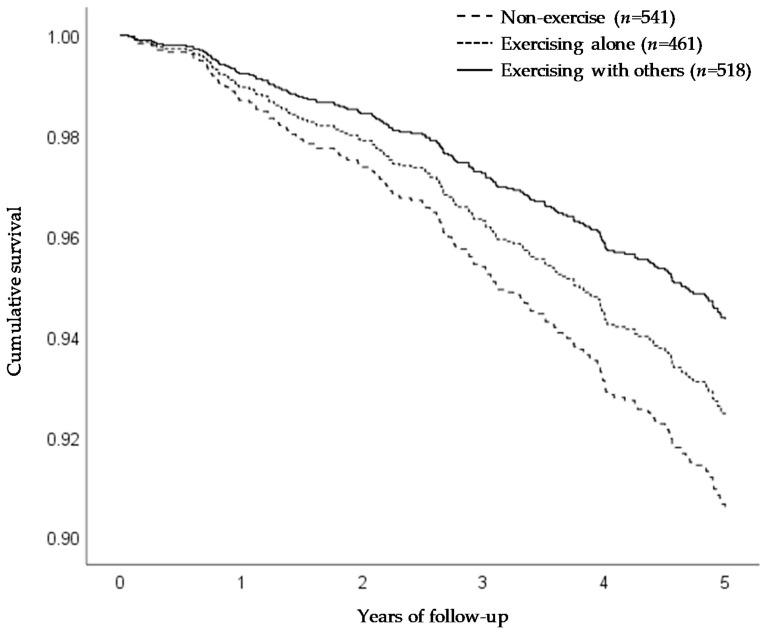
The adjusted Kaplan–Meier curves for functional disability by exercise habits among all subjects in model 2. Survival curves were adjusted for age, gender, body mass index, living arrangement, subjective economic condition, medical history, fall history, and depressive symptoms.

The relationship between exercise habits and functional disability is shown in Table 2. During a mean follow-up of 4.7 years (7169 person-years), functional disability developed in 185 subjects (12.2%). Compared with the non-exercise group, exercising with others significantly lowered the risk of incident functional disability in all models. Compared with exercising alone, exercising with others decreased the risk of incident functional disability in the unadjusted model. However, no significant difference was noted between those who exercised alone and those who exercised with others in model 1 (*p* = 0.06) and model 2 (*p* = 0.17). Figure 1 shows the adjusted Kaplan–Meier curves for functional disability by exercise habits among all subjects in model 2.

**Figure 2:**
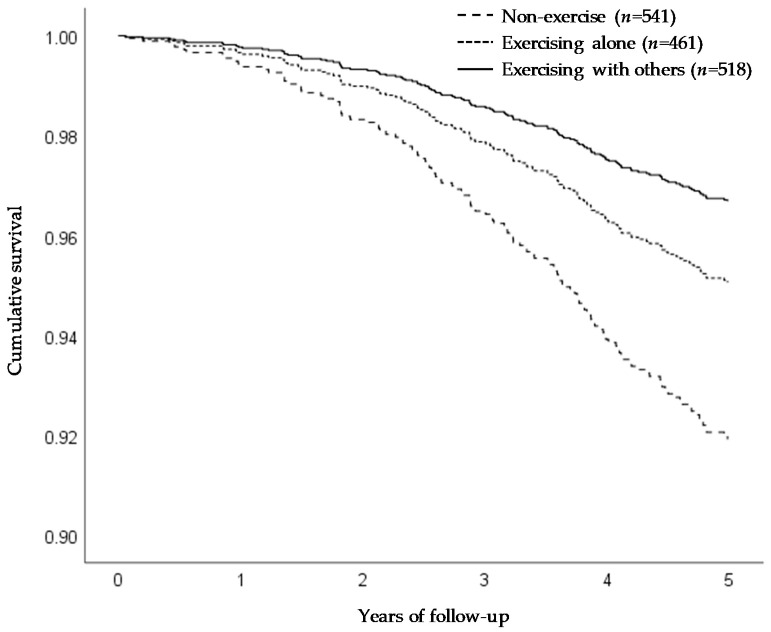
Adjusted Kaplan–Meier curves for morality by exercise pattern. Survival curves were adjusted for age, gender, body mass index, living arrangement, subjective economic condition, medical history, fall history, and depressive symptoms.

Table 3 and Figure 2 show the results for all-cause mortality. During a mean follow-up of 4.8 years (7328 person-years), 128 deaths (8.4%) occurred. Compared with non-exercise, both exercising alone and exercising with others were associated with a significantly lower mortality rate in the unadjusted model, and these associations remained statistically significant after adjusting for potential confounders, though the HRs were slightly changed. Compared with exercising alone, exercising with others was associated with a lower mortality rate in the unadjusted model; however, no significant difference was noted between those who exercised alone and those who exercised with others in models 1 (*p* = 0.08) and 2 (*p* = 0.16).

## 4. Discussion

This study examined the association of exercise habits with functional disability and all-cause mortality for five years among community-dwelling older adults. As we hypothesized, older adults who exercised with others had a lower risk of incident functional disability and mortality than those who did not exercise. Additionally, compared with exercising alone, exercising with others was more likely to decrease the risk of functional disability and mortality, though no statistically significant differences were observed in the covariate models. These findings suggest that social aspects of exercise are important for a long, healthy life among older adults.

Our results showed that exercising with others helped prevent functional disability, but not exercising alone. Similarly, a previous study reported that joining a sports organization correlated with a lower risk of incident functional disability for four years than exercising alone [12]. However, that study had a methodological limitation in that no clear distinction between those who exercised alone and those who exercised with others was made. Our study supports and expands these previous findings by showing the benefits of exercising with others, not limited to participation in a group, in terms of the prevention of functional disability among community-dwelling older adults.

In this study, we were able to confirm a favorable relationship between exercise habits and mortality. Compared with those who did not exercise, older adults who either exercised alone or exercised with others had a lower mortality rate for five years, despite the adjustment of the analysis for potential confounders. Conversely, we did not observe a statistically significant difference between exercising alone and exercising with others in models 1 and 2. This may be attributable to the low statistical power of our sample to detect a difference in mortality between the two groups because the HRs tended to be relatively low and we could see the significant difference in the unadjusted model and the *p* value was <0.1 in model 1. Thus, a more robust sampling, longer follow-up period, and/or a larger sample size may allow detection of such associations. 

Although there were nonsignificant differences between the two exercise groups in the covariate models, we confirmed the trend for a decrease in the risk of functional disability and mortality in older adults who were exercising with others, and this finding is consistent with those in previous studies. According to the Japan Gerontological Evaluation Study, individuals who exercised with others are related with a better subjective health status [9] and lower incidence of falls in the previous year [10]. Another longitudinal study showed that exercising with others has the potential to decrease the risk of depression [8], which is a known important predictor of mortality [16]. In addition, a review on the benefits of group exercise highlighted that exercising with others is associated with three factors in terms of additional health benefits, including social, psychological, and quantitative factors [17]. Particularly, social factors enhancing social connectedness may have a large effect on health because social connectedness is independently related to several health benefits, including decreased mortality [18]. In the present study, although the underlying mechanism could not be determined, the above factors may have influenced the trend of the decrease in functional disability and mortality. 

In this rapidly aging society, promoting exercise as a way to achieve successful aging and longevity is gaining attention. However, at present, only the quantitative aspects of exercise, rather than the qualitative aspects, have been emphasized in health guidelines and policies. In other words, “*What should we do?*” is a more common issue than “*How should we do?*”. Our study attempted to confirm the effect of the social aspects of exercise and revealed certain health benefits of exercising with others. To establish an effective strategy of utilizing the benefits of exercising with others, further large and long prospective cohort studies are warranted. 

The strength of this study was related to its five-year follow-up of older adults and investigation of the effect of exercising with others on robust endpoints, including death, for the first time. This study, however, had some limitations. First, the exercise frequency, duration, intensity, and amount of physical activity could not be evaluated in this study. Exercising with groups might prolong the duration of exercise [19] and help meet the required amount of physical activity [11]. Hence, these aspects might have mediated (confounded) the associations investigated in this study, regardless of social aspects. Second, the reliability and validity of the measurement of exercise habits have not been confirmed, while the question was the same as the previous study [11]. Because the self-reported exercise habits may differ from actual exercise behavior, the results may have contained some measurement bias. Third, although potential confounders at baseline were used as covariates in the analysis, we could not fully exclude the confounding effects of other factors such as educational status, smoking habits, or alcohol consumption. Since the individual who participated in a sports group tended to have higher socioeconomic status [20], we should have investigated and controlled these confounders. Fourth, due to the sample size, it was difficult to stratify the analysis by sex, age, and health level. Particularly, gender differences will be considered in the future. Fifth, data regarding the reason of death were not obtained; this information is important to understand the specific effects of exercising with others. Finally, although the study was a complete survey, there were some non-responders or missing data. Thus, some selection bias may have existed, and the results should be carefully generalized.

## 5. Conclusions

In conclusion, this study revealed that exercising with others would help decrease the risk of functional disability and all-cause mortality in older adults. Our findings suggested the importance of the social aspects of exercise in the promotion of public health and longevity.

## Figures and Tables

**Table 1:** Baseline characteristics of participants according to exercise habits.

Baseline Variables	Non-Exercise		Exercising Alone		Exercising with Others		*p*-Value
No. of participants	541		461		518		
Mean (SD) age (years)	73.9	(6.8)	73.5	(6.5)	72.9	(5.5)	0.02
Male sex	284	(52.5)	240	(52.1)	217	(41.9)	<0.01
BMI (kg/m^2^)							0.49
<18.5	28	(5.2)	28	(6.1)	21	(4.1)	
18.5–29.9	502	(92.8)	425	(92.2)	491	(94.8)	
≥30	11	(2.0)	8	(1.7)	6	(1.2)	
Living alone	60	(11.1)	54	(11.7)	48	(9.3)	0.43
Poor economic condition	112	(20.7)	92	(20.0)	84	(16.2)	0.14
Stroke	24	(4.4)	19	(4.1)	20	(3.9)	0.90
Heart diseases	90	(16.6)	61	(13.2)	59	(11.4)	0.04
Joint pain	93	(17.2)	74	(16.1)	103	(19.9)	0.27
Fall history	136	(25.1)	97	(21.0)	92	(17.8)	0.01
Mean (SD) Depressive symptoms (points)	1.0	(1.4)	0.8	(1.2)	0.5	(0.9)	<0.01

Values are numbers (percentages) unless stated otherwise. SD, standard deviations. The *p*-value for differences between groups calculated using chi-squared tests for categorical variables and analysis of variance for continuous variables.

**Table 2:** Hazard ratios for the five-year incident functional disability according to exercise habits.

Variables	Hazard Ratio (95% CI)				
	Non-Exercise	Exercising Alone		Exercising with Others	
No. of participants	541	461		518	
No. of person-years	2479	2175		2515	
No. of incidence	88	60		37	
Incidence rates per 1000 person-years	36	28		15	
All subjects					
Unadjusted	1.00	0.78	(0.59–1.08)	0.41	(0.28–0.61)
Model 1 ^†^	1.00	0.81	(0.59–1.13)	0.54	(0.37–0.80)
Model 2 ^‡^	1.00	0.80	(0.57–1.11)	0.59	(0.40–0.88)
Only exercisers					
Unadjusted		1.00		0.53	(0.35–0.80)
Model 1 ^†^		1.00		0.67	(0.44–1.02)
Model 2 ^‡^		1.00		0.74	(0.48–1.14)

^†^ Adjusted for age and sex. ^‡^ Additional adjustment of model 1 for body mass index, living arrangement, subjective economic condition, medical history, fall history, and depressive symptoms. CI, confidence interval.

**Table 3:** Hazard ratios for five-year mortality by exercise habits.

Variables	Hazard Ratio (95% CI)				
	Non-Exercise	Exercising Alone		Exercising with Others	
No. of participants	541	461		518	
No. of person-years	2546	2237		2545	
No. of death	70	37		21	
Incidence rates per 1000 person-years	28	17		8	
All subjects					
Unadjusted	1.00	0.60	(0.40–0.89)	0.31	(0.18–0.48)
Model 1 ^†^	1.00	0.61	(0.41–0.90)	0.37	(0.23–0.61)
Model 2 ^‡^	1.00	0.60	(0.40–0.90)	0.40	(0.24–0.66)
Only exercisers					
Unadjusted		1.00		0.50	(0.29–0.85)
Model 1 ^†^		1.00		0.62	(0.36–1.06)
Model 2 ^‡^		1.00		0.67	(0.38–1.17)

^†^ Adjusted for age and sex. ^‡^ Additional adjustment of model 1 for body mass index, living arrangement, subjective economic condition, medical history, fall history, and depressive symptoms. CI, confidence interval.
